# ﻿*Paraphlomisyingdeensis* (Lamiaceae), a new species from Guangdong (China)

**DOI:** 10.3897/phytokeys.219.97547

**Published:** 2023-02-08

**Authors:** Guo-Xin Guo, Wan-Yi Zhao, Ya-Ping Chen, Jin-Hai Xiao, Yuan-Qiu Li, Qiang Fan

**Affiliations:** 1 Guangdong Shimentai National Nature Reserve, Yingde 513000, China Guangdong Shimentai National Nature Reserve Yingde China; 2 State Key Laboratory of Biocontrol and Guangdong Provincial Key Laboratory of Plant Resources, School of Life Sciences Sun Yat-Sen, Sun Yan-Sen University, Guangzhou 510275, China Sun Yan-Sen University Guangzhou China; 3 CAS Key Laboratory for Plant Diversity and Biogeography of East Asia, Kunming Institute of Botany, Chinese Academy of Science, Kunming 650201, China Kunming Institute of Botany, Chinese Academy of Science Kunming China

**Keywords:** endemics, limestone, new taxon, Paraphlomideae, phylogeny

## Abstract

*Paraphlomisyingdeensis* (Lamiaceae), a new species from the limestone area in northern Guangdong Province, China, is described and illustrated. Phylogenetic analyses, based on two nuclear DNA regions (ITS and ETS) and three plastid DNA regions (*rpl32-trnL*, *rps16* and *trnL-trnF*), suggest that *P.yingdeensis* represents a distinct species in *Paraphlomis*. Morphologically, *P.yingdeensis* is similar to P.foliatasubsp.montigena and *P.nana*, but can be distinguished from the former by its densely villous lamina and calyx, not decurrent base of lamina and bristle-like-acuminate apex of calyx teeth, and distinguished from the latter by its significantly taller plant (15–20 cm vs. 1–5 cm) and larger lamina (6.2–16.5 × 4–11.5 vs. 2–7 × 1.5–4 cm), densely villous stem, lamina and calyx and yellow corolla.

## ﻿Introduction

As a member of tribe Paraphlomideae (Lamiaceae, Lamioideae) (Bendiksby et al. 2011; Zhao et al. 2021), the genus *Paraphlomis* Prain is characterised by its herbaceous habit, actinomorphic calyx with five lobes less than half as long as the tube, corolla 2-lipped (1/3) with hairy upper lip, but hardly bearded along the margin, included stamens and an apically truncate ovary (Wu and Li 1977; Bendiksby et al. 2011; Chen et al. 2021). A total of 36 species and seven varieties are recognised within *Paraphlomis*, most of which are distributed in southern China (Chen et al. 2022b; Yuan et al. 2022), with several species occurring in the Himalayas, Korea and Southeast Asia (Li and Hedge 1994; Ko et al. 2014; Chen et al. 2021). Many species of *Paraphlomis* are endemics of limestone soils, including the recently described *P.kuankuoshuiensis* R.B. Zhang, D. Tan & C.B. Ma (Zhang et al. 2020), *P.longicalyx* Y.P. Chen & C.L. Xiang (Chen et al. 2022a) and *P.hsiwenii* Y.P. Chen & X. Li (Chen et al. 2022b). This shows species richness of *Paraphlomis* has been quite underrated and more field investigations are needed to infer its diversity in limestone areas.

The botanical expedition to the Shimentai National Nature Reserve in Guangdong Province, China in October 2021, showed an unknown species of *Paraphlomis*. Based on other field observations (from May to August in 2022), morphological comparisons with congeneric species, as well as molecular phylogenetic studies, we confirmed that it represented a new species, here described and illustrated.

## ﻿Materials and methods

### ﻿Morphological study

Field observations and collections of the new species were carried out from May to August in 2022 in Boluo Town of Yingde City in northern Guangdong Province, China. Morphological comparisons of the putative new species with other *Paraphlomis* species were conducted firstly by consulting relevant taxonomic literature, included “Flora of China” (Li and Hedge 1994), “Flora of Guangdong” (Luo 1995) and other recently described species and infraspecies of *Paraphlomis* (Yan and Fang 2009; Ding et al. 2019; Zhang et al. 2020; Chen et al. 2021, 2022a, b, c; Zhao et al. 2022). We also carried out a check of herbarium specimens deposited in LBG, AU, IBK, FJFC, PE, ANUB, KUN, FJSI and SYS (herbarium acronyms following Thiers 2022). All morphological characters were measured using dissecting microscopes.

### ﻿Phylogenetic analyses

Previous molecular phylogenetic study revealed genus *Paraphlomis* is not monophyletic, because species of *Matsumurella* were recovered within it (Chen et al. 2021; Chen et al. 2022b). Thus, *Matsumurella* was also included in our phylogenetic analyses. A total of 37 accessions, representing 20 species and four varieties/subspecies of *Paraphlomis* and two *Matsumurella* species were selected as ingroups. One species each of *Phlomis* L. and *Phlomoides* Moench were included as outgroups following Chen et al. (2022a, b). Except for the three accessions of the new species that were newly sampled here, sequences of the remaining accessions were all retrieved from our previous studies (Chen et al. 2021, 2022a, b, c). Genomic DNA of the potential new species was extracted from silica-gel-dried leaves using the modified 2× CTAB procedure of Doyle and Doyle (1987). We selected five DNA markers for the phylogenetic reconstruction, including two nuclear ribosomal regions (internal and external transcribed spacers, i.e. ITS and ETS) and three plastid DNA regions (*rpl32-trnL*, *rps16* and *trnL-trnF*). Primers used for the polymerase chain reaction (PCR) amplification and sequencing were the same as those of Chen et al. (2021), while PCR procedures followed those described in Chen et al. (2016). The specimen information of samples and GenBank accession numbers for all sequences are listed in Appendix 1.

Raw sequences were assembled and edited using Sequencher 4.1.4 (Gene Codes, Ann Arbor, MI, USA) and then aligned using MUSCLE (Edgar 2004) and manually adjusted in MEGA 6.0 (Tamura et al. 2013). Bayesian Inference (BI) (Ronquist et al. 2012) and Maximum Likelihood (ML) (Stamatakis 2014) analyses were used for phylogenetic reconstruction and detailed settings for the two analyses followed those described in Chen et al. (2021). The resulting trees with posterior probabilities (PP) and Bootstrap support (BS) values were visualised and annotated in TreeGraph 2 (Stöver and Müller 2010). The combined nuclear dataset and the combined plastid dataset were initially analysed separately. Topological incongruence between the two reconstructions was visually inspected, based on the thresholds of PP ≥ 0.95 and/or BS ≥ 70%. After excluding the taxa that exhibited strong conflicts between the nuclear tree and the plastid tree, the combined nuclear dataset and the combined plastid dataset were then concatenated for phylogenetic analyses.

## ﻿Results and discussion

**Figure 1.**
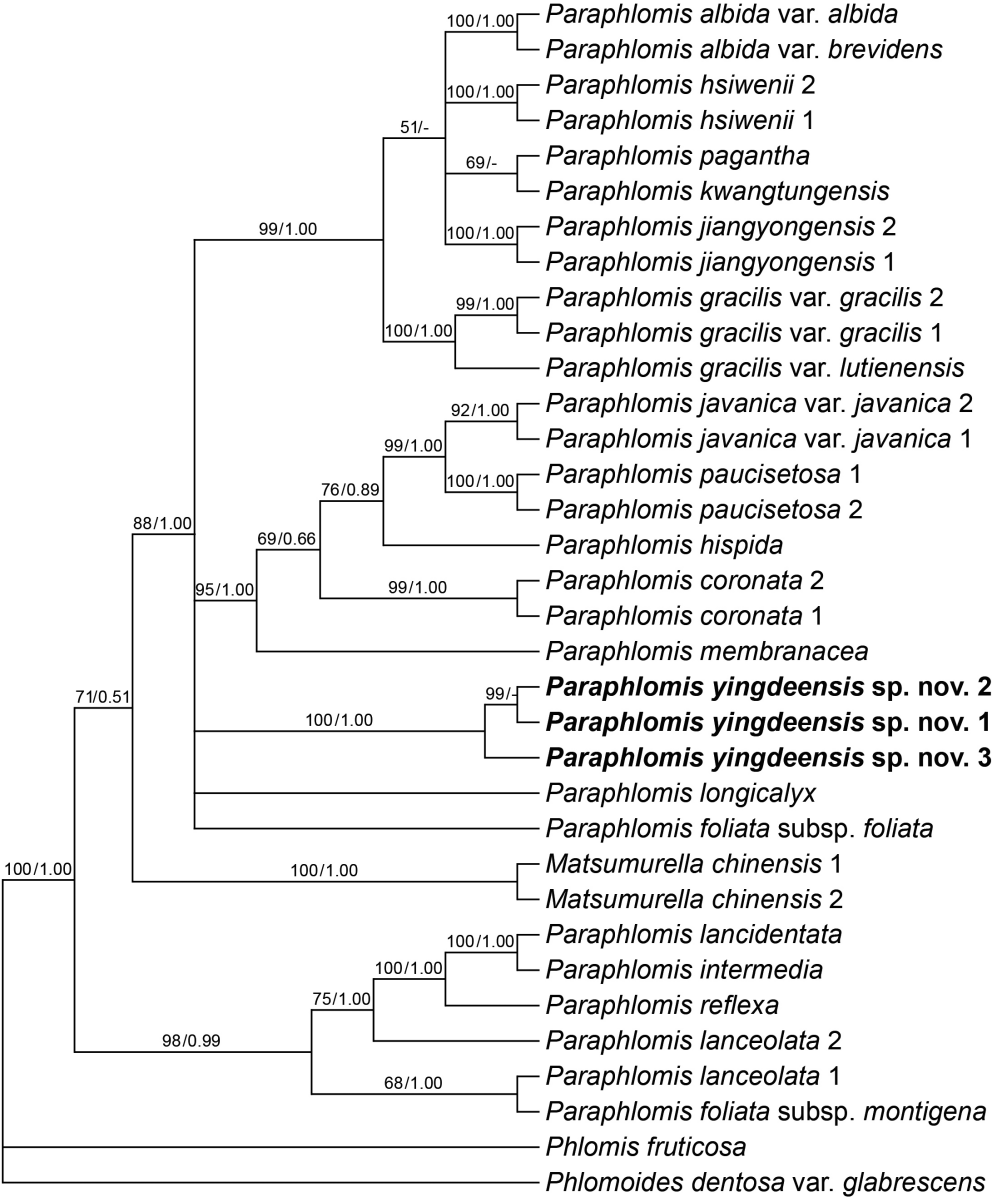
Optimal Maximum Likelihood tree of *Paraphlomis* inferred from combined nuclear (ETS and ITS) and plastid (*rpl32-trnL*, *rps16* and *trnL-trnF*) dataset. Support value ≥ 50% BS or 0.50 PP are displayed above the branches (“-” indicates a support value < 0.50 PP).

The aligned length of the combined nuclear dataset was 1254 bp (810 bp for ITS, 444 bp for ETS) and that of the combined plastid dataset was 2479 bp (850 bp for rpl32-trnL, 812 bp for rps16, 817 bp for trnL-trnF). Since the placements of three taxa, *Paraphlomisalbiflora* (Hemsl.) Hand.-Mazz., *P.nana* Y.P. Chen, C. Xiong & C.L. Xiang and P.javanicavar.pteropoda D. Fang & K.J. Yan, showed hard incongruences in the nuclear tree (Appendix 2) and the plastid tree (Appendix 3), these taxa were excluded prior to the combination of the nuclear and plastid datasets. All the resulting trees (Fig. 1; Appendices 2–3) were topologically consistent with those in previous study (Chen et al. 2021). With the two species of *Matsumurella* deeply nested within *Paraphlomis*, both genera were shown to be non-monophyletic. The three individuals of the putative new species formed a strongly supported clade (Fig. 1: PP = 1.00 / BS = 100%), but its relationship with other species of *Matsumurella*-*Paraphlomis* was not resolved.

Our morphological study revealed that the new species *P.yingdeensis* W.Y.Zhao, Y.Q.Li & Q.Fan is most similar to P.foliatasubsp.montigena X.H. Guo & S.B. Zhou and *P.nana* for some morphological characters as they have short habits and triangular-laceolate calyx teeth with apices acuminate or bristle-like-acuminate. Paraphlomisfoliatasubsp.montigena was classified by Guo (1993) as a subspecies of *P.foliata* (Dunn) C.Y. Wu & H.W. Li. However, previous molecular phylogenetic studies (Chen et al. 2022b, c) and our present analyses (Fig. 1; Appendices 2–3) indicated that P.foliatasubsp.montigena might represent an independent species within the genus as it is distantly related to P.foliatasubsp.foliata. The new species can be distinguished from P.foliatasubsp.montigena in the morphology and indumentum of laminae and calyces. Both the laminae and calyces are densely villous in *P.yingdeensis*, but are sparsely strigose in P.foliatasubsp.montigena; the base of lamina is broadly cuneate and not decurrent in the new species, but is cuneate and decurrent in P.foliatasubsp.montigena; *P.yingdeensis* has bristle-like-acuminate apex of calyx teeth, in contrast, the apex of calyx teeth of P.foliatasubsp.montigena is acuminate. The phylogenetic placement of *P.nana* was conflicting in the nuclear tree and plastid tree, but it was consistently sister to *P.albiflora* (Appendices 2–3). Both *P.nana* and *P.yingdeensis* have translucent and membranous calyces with bristle-like-acuminate apex of calyx teeth. The two species mainly differ in the height of plants, size and indumentum of laminae, as well as colour of corollae. Specifically, plants of *P.nana* are 1–5 cm tall, whereas those of *P.yingdeensis* are 10–20 cm tall. The stems and laminae are densely villous in *P.nana*, but are densely strigose in *P.yingdeensis*. Moreover, *P.yingdeensis* has significantly larger laminae than *P.nana* (6.2–16.5 × 4–11.5 cm vs. 2–7 × 1.5–4 cm) and the corollae of *P.yingdeensis* are yellow, differing from the white corollae of *P.nana*. Detailed morphological comparisons amongst the three taxa were summarised in Table 1.

**Table 1.** Morphological comparisons amongst *Paraphlomisyingdeensis*, P.foliatasubsp.montigena and *P.nana*.

Characters	*P.yingdeensis*	P.foliatasubsp.montigena	*P.nana*
**Stem**	10–20 cm tall, densely villous	15–20 cm tall, densely villous	1–5 cm tall, densely retrorse strigose
**Lamina**	6.2–16.5 × 4–11.5 cm, base broadly cuneate, not decurrent, densely villous	5–16 × 2.5–6.5 cm, base cuneate, decurrent, sparsely strigose	2–7 × 1.5–4 cm, base cuneate to broadly cuneate, decurrent, densely to sparsely strigose
**Calyx**	Densely villous outside, teeth 3–4 mm long, apex bristle-like-acuminate	Sparsely strigose outside, teeth ca. 2.5 mm long, apex acuminate	Appressed strigose outside, teeth ca. 3 mm long, apex bristle-like-acuminate
**Corolla**	yellow	yellow	white

Geographically, P.foliatasubsp.montigena is restricted to the Qingliangfeng Nature Reserve at the border area of Zhejiang and Anhui Provinces in eastern China (Guo 1993) and *P.nana* is now only known from Chongqing City in central China (Chen et al. 2022c). Both the two species are not karst-adapted. In contrast, the new species is distributed in the limestone area in Guangdong Province, southern China.

### ﻿Taxonomic treatment

#### ﻿ Paraphlomis yingdeensis

Taxon classificationPlantaeLamialeLamiaceae

W.Y.Zhao, Y.Q.Li & Q.Fan sp. nov.

2B9BE82B-19BE-5389-B061-A4A45A2AFD06

urn:lsid:ipni.org:names:77313387-1

**Figure 2.**
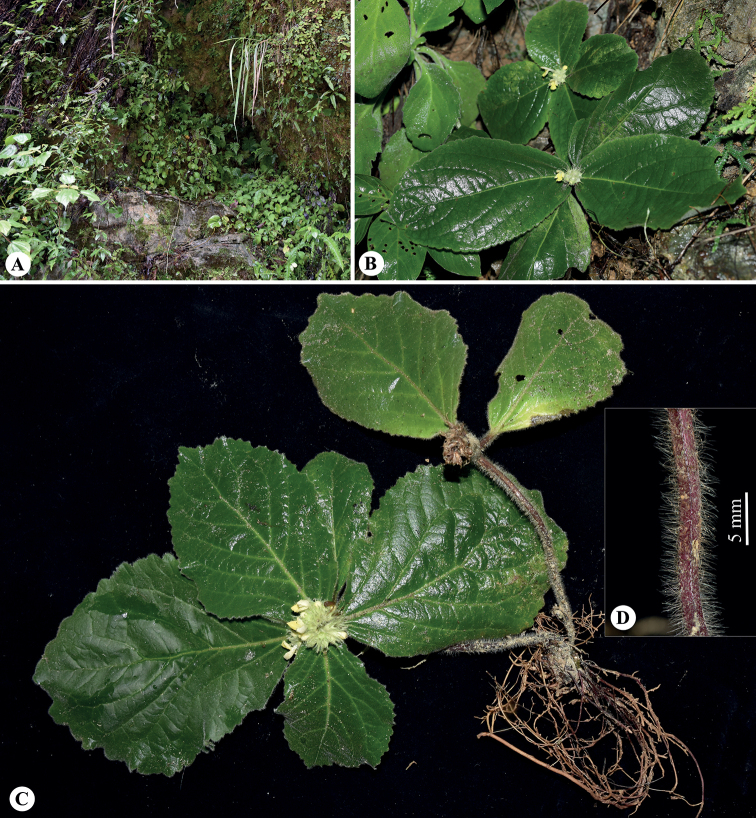
*Paraphlomisyingdeensis* from the type locality **A** habitat **B, C** plants **D** stem. (Photographs: **A, C, D** by W.-Y. Zhao; **B** by Y.-Q. Li).

**Figure 3.**
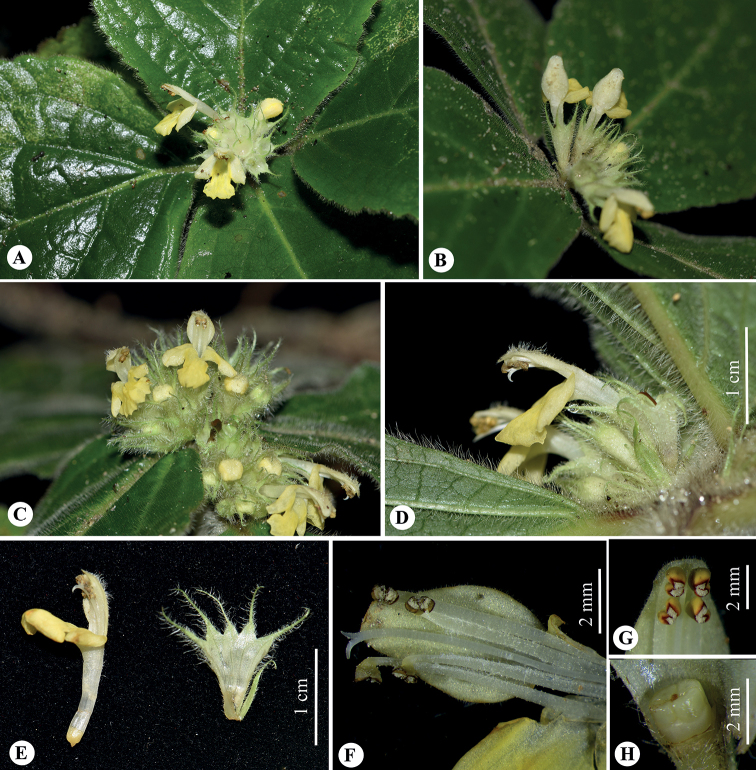
Floral traits of *Paraphlomisyingdeensis***A, B** inflorescences **C** frontal view of flower **D** lateral view of flower **E** corolla and dissected calyx (inner view) **F** pistil and stamens **G** anthers **H** ovary. (Photographs: **A, B** by Y.-Q. Li; **C–H** by W.-Y. Zhao).

**Figure 4.**
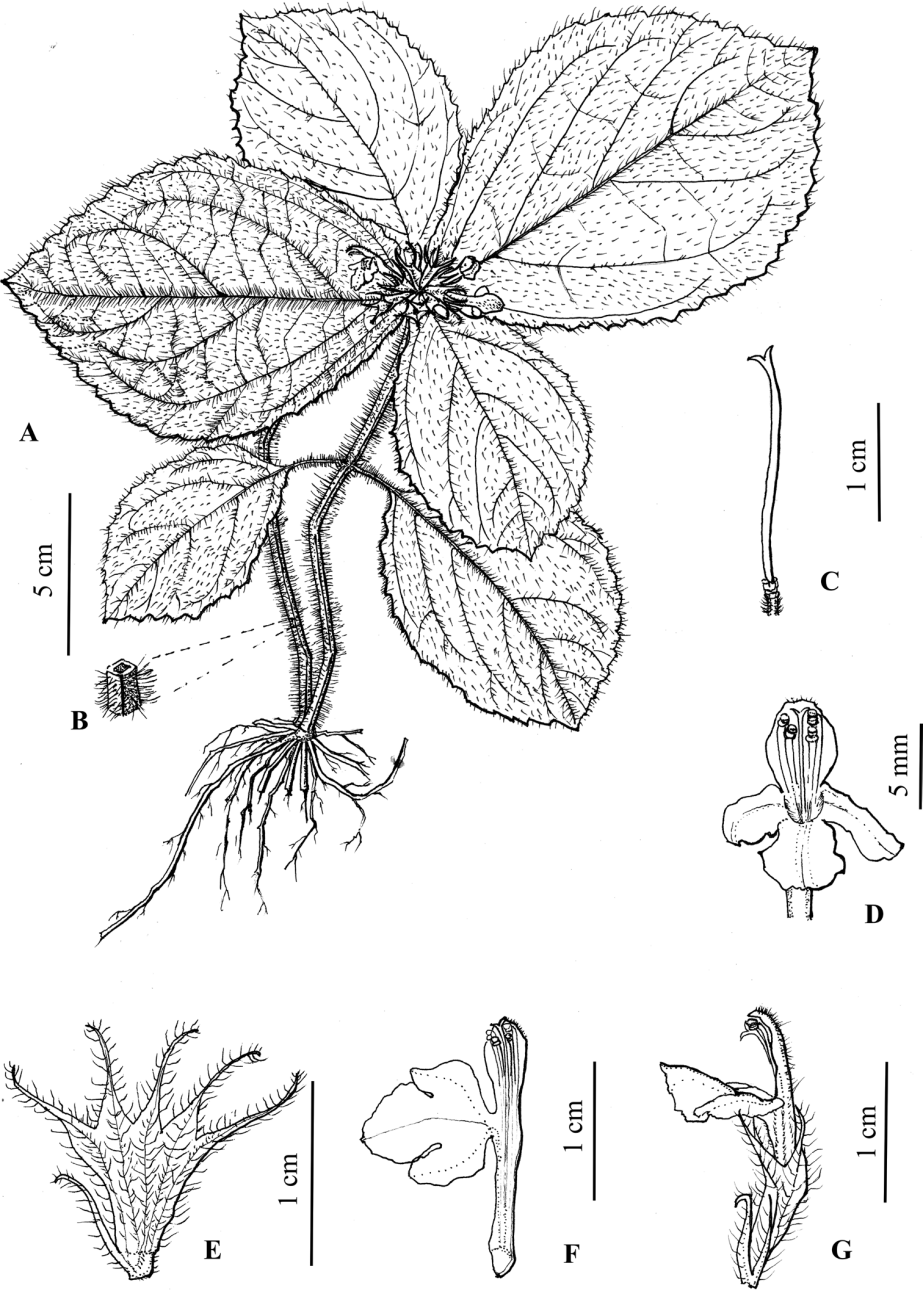
Line drawing of *Paraphlomisyingdeensis***A** plant **B** transverse section of stem **C** pistil **D** frontal view of flower **E** dissected calyx (outside view) **F** dissected corolla **G** lateral view of flower. (Drawn by Rong-En Wu).

Figs 2 , 3 , 4 Chinese name: 英德假糙苏

##### Type.

China. Guangdong Province: Yingde City, Boluo Town, on the way from Xinzhai Village to Changshan Village, on the limestone cliff at the roadside, 24°24'N, 113°0'E, alt. 61 m, 29 May 2021, *Zhao Wan-Yi*, *Li Yuan-Qiu*, *Pan Jia-Wen & Yang Ling-Han ZWY-2092* (holotype: SYS00236856! isotypes: KUN!, SYS00236857!, SYS00236858!, SYS00236859!)

##### Diagnosis.

*Paraphlomisyingdeensis* is morphologically similar to P.foliatasubsp.montigena and *P.nana*, but differs from the former in its lamina and calyx densely villous (vs. sparsely strigose), base of lamina not decurrent (vs. decurrent) and apex of calyx teeth bristle-like-acuminate (vs. acuminate) and from the latter in its plants 10–20 cm tall (vs. 1–5 cm tall), lamina 6.2–16.5 × 4–11.5 cm and densely villous (vs. 2–7 × 1.5–4 cm and densely strigose) and corolla yellow (vs. white).

##### Description.

***Herbs*** perennial, 10–20 cm tall. ***Rhizomes*** short; roots fibrous, yellowish-brown, glabrous. ***Stems*** erect or prostrate, 4-angled, green (young branch) to purplish-red, densely villous. ***Leaves*** opposite, leafless towards base, upper two pairs crowded and rosulate; petiole 0.3–2.5 cm long, densely villous; lamina obovate, papery, 6.2–16.5 cm long, 4–11.5 cm wide, apex obtuse, base broadly cuneate, margin crenate-serrate; adaxially green, abaxially light green, densely villous on both sides; lateral veins 5–7-paired, obviously raised abaxially. ***Verticillasters*** in compact, sometimes capitate-like inflorescences, 8–16-flowered, 2.2–3.0 cm in diam.; bracteoles lanceolate to linear, 7–8 mm long, densely villous. ***Calyx*** light green, translucent, membranous, campanulate, 6–7 mm long, densely villous outside, glabrous inside, conspicuously 10-veined; teeth 5, subequal, triangular lanceolate, 3–4 mm long, apex bristle-like-acuminate. ***Corolla*** yellow, 1.5–1.8 cm long; tube 1.0–1.1 cm long, ca. 1.5 mm in diam., straight, pubescent annulate in throat inside; 2-lipped, villous outside, upper lip oblong, margin entire, erect, ca. 6 mm long, ca. 3.5 mm wide; lower lip spreading or reflexed, 4–5 mm long, 3-lobed, medium lobe suborbicular, apex emarginate, lateral lobes oblong, apex obtuse. ***Stamens*** 4, inserted above middle and upper of corolla tube, straight, included, filaments flat, sparsely puberulent-villous; anther cells 2, ovoid, glabrous. ***Style*** filiform, included, glabrous, apex subequally 2-lobed. ***Ovary*** 4-loculed, glabrous. ***Nutlets*** not seen.

##### Distribution and habitat.

Currently, only one population of *P.yingdeensis* was found in Boluo Town, Yingde City, in northern Guangdong Province. This town was located in the subtropical monsoon climate region, with development of a large area of karst landform. *Paraphlomisyingdeensis* usually grows on moist limestone cliffs in evergreen broad-leaved forests in association with *Tectariadevexa* Copel., *Primulinayingdeensis* Z.L. Ning, M. Kang & X.Y. Zhuang, *Begonialeprosa* Hance and *Ficus* spp.

##### Phenology.

Flowering from May to June and fruiting from June to August.

##### Etymology.

The specific epithet “*yingdeensis*” is derived from the type locality of the new species, i.e. Yingde City in Guangdong, China.

##### Additional specimens examined

(**paratypes**). China. Guangdong Province: Yingde City, Boluo Town, on the way from Xinzhai Village to Changshan Village, 24°24'N, 113°0'E, alt. 61 m, 9 June 2021, *Q. Fan 19013* (SYS); *ibid.*, 5 June 2022, *Li Yuan-Qiu ZWY-2020* (SYS); *ibid.*, 14 August 2022, *Ye Fan ZWY-2032* (SYS).

##### Specimens of P.foliatasubsp.montigena examined.

China. Anhui Province: Xi County, Qingliangfeng, alt. 1300 m, 29 October 1980, *Guo Xin-Hu 800023* (ANUB 13030926); *ibid.*, 16 July 1989, *Guo Xin-Hu & Zhou Shou-Biao 89011* (KUN 778733).

##### Specimens of *P.nana* examined.

China. Chongqing: Chongkou County, Mingzhong Town, Jinchi Village, Longmenxi, Dabashan National Natural Reserve, on the moist cliff, alt. 996 m, 7 July 2021, *Chi Xiong XC21097* (holotype: KUN; isotypes: CQNM, IBK); Wushan County, Zhuxian Town, Shizhuzi Village, Daguling, Wulipo National Natural Reserve, in the moist valley, alt. 1310 m, 18 July 2021, *Chi Xiong & Hou-Lin Zhou XC21126* (KUN); *ibid.*, 11 September 2021, *Hou-Lin Zhou s.n.* (KUN).

## Supplementary Material

XML Treatment for Paraphlomis yingdeensis

## Figures and Tables

**Figure A1.**
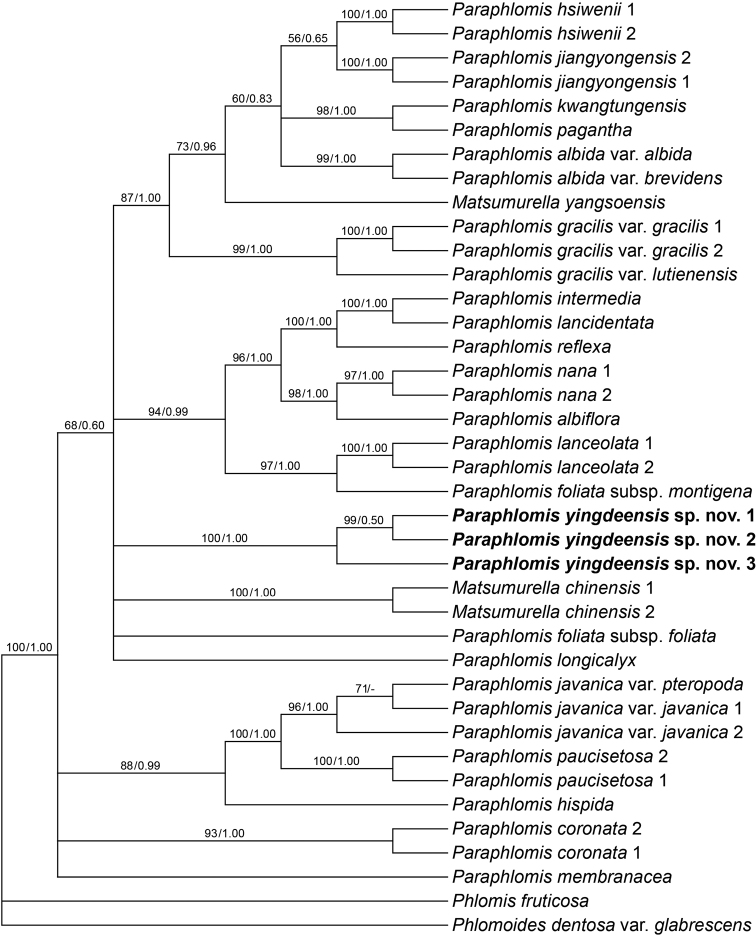
Optimal Maximum Likelihood tree of *Paraphlomis* inferred from combined nuclear (ITS and ETS) dataset. Support value ≥ 50% BS or 0.50 PP are displayed above the branches (“-” indicates a support value < 0.50 PP).

**Figure A2.**
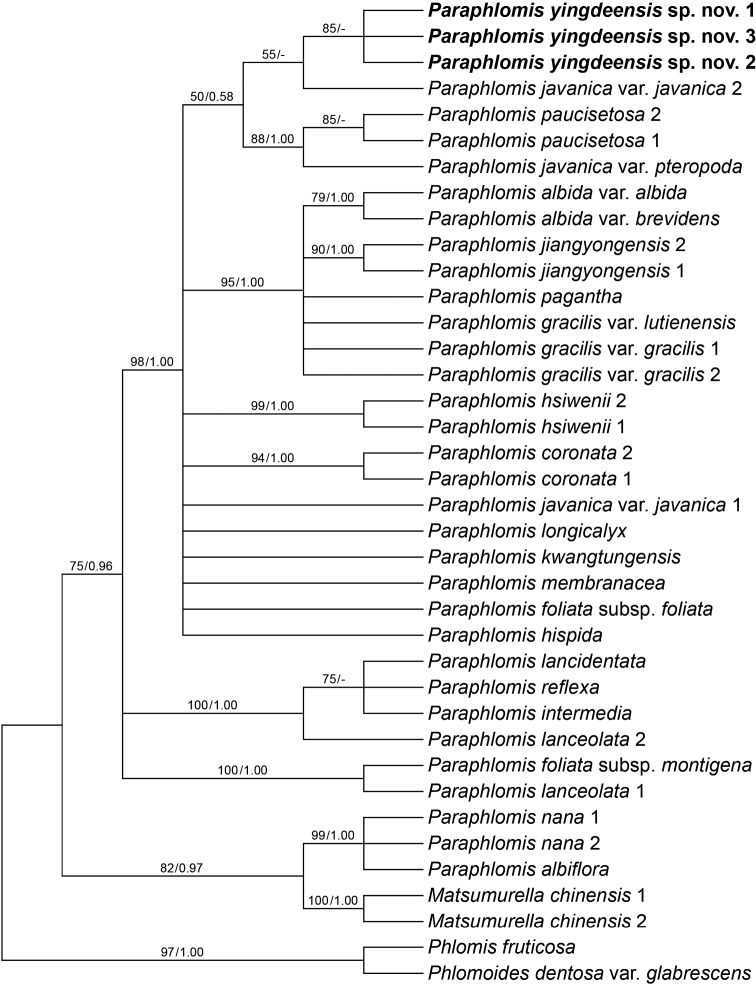
Optimal Maximum Likelihood tree of *Paraphlomis* inferred from combined plastid (*rpl32-trnL*, *rps16* and *trnL-trnF*) dataset. Support value ≥ 50% BS or 0.50 PP are displayed above the branches (“-” indicates a support value < 0.50 PP).

## ﻿Appendix 1

**Table A1.** Sequence information for all samples used in present study. A “/” indicates a missing sequence. Herbarium abbreviations are listed after the vouchers. The accession numbers marked in bold represent sequences newly generated.

Taxon	Voucher	Country	ITS	ETS	*rpl32-trnL*	*rps16*	*trnL-trnF*
*Matsumurellachinensis* (Benth.) Bendiksby 1	Y. Yang OYY00316 (KUN)	Pingxiang, Jiangxi, China	MW602147	MW602117	MW602021	MW602053	MW602084
*Matsumurellachinensis* (Benth.) Bendiksby 2	Y. Yang OYY00131 (KUN)	Guilin, Guangxi, China	MW602148	MW602118	MW602022	MW602054	MW602085
*Matsumurellayangsoensis* (Y.Z. Sun) Bendiksby	L. Wu & W.B. Xu 10965 (IBK)	Yangshuo, Guangxi, China	MW602142	MW602112	/	/	/
ParaphlomisalbidaHand.-Mazz.var.albida	A. Liu et al. LK0841 (CSFI)	Ningyuan, Hunan, China	MW602124	MW602091	MW601996	MW602028	MW602060
Paraphlomisalbidavar.brevidens Hand.-Mazz.	Y.P. Chen EM312 (KUN)	Hezhou, Guangxi, China	MW602130	MW602098	MW602003	MW602035	MW602067
*Paraphlomisalbiflora* (Hemsl.) Hand.-Mazz.	C.M. Tan et al. 1806393 (JJF)	Jiujiang, Jiangxi, China	/	MW602101	MW602006	MW602038	MW602069
*Paraphlomiscoronata* (Vaniot) Y.P. Chen & C.L. Xiang 1	E.D. Liu et al. 3043 (KUN)	Emeishan, Sichuan, China	MW602137	MW602107	MW602012	MW602044	MW602075
*Paraphlomiscoronata* (Vaniot) Y.P. Chen & C.L. Xiang 2	C.L. Xiang 358 (KUN)	Jiangkou, Guizhou, China	MW602123	MW602090	MW601995	MW602027	MW602059
Paraphlomisfoliata(Dunn)C.Y. Wu & H.W. Lisubsp.foliata	S.P. Chen s.n. (KUN)	Jiangle, Fujian, China	/	MW602097	MW602002	MW602034	MW602066
Paraphlomisfoliatasubsp.montigena X.H. Guo & S.B. Zhou	Y.C. Dai s.n. (KUN)	Hangzhou, Zhejiang, China	OM836064	OM884453	OM884456	OM884459	OM884462
Paraphlomisgracilis(Hemsl.) Kudôvar.gracilis 1	A. Liu LK0931 (CSFI)	Changsha, Hunan, China	MW602134	MW602104	MW602009	MW602041	MW602072
Paraphlomisgracilis(Hemsl.) Kudôvar.gracilis 2	C.L. Xiang XCL1315 (KUN)	Chongqing, China	MW602141	MW602111	MW602016	MW602048	MW602079
Paraphlomisgracilisvar.lutienensis (Y.Z. Sun) C.Y. Wu	C.L. Xiang XCL881 (KUN)	Shibing, Guizhou, China	MW602131	MW602099	MW602004	MW602036	MW602068
*Paraphlomishispida* C.Y. Wu	X. Li LX200702 (GXF)	Napo, Guangxi, China	MW602132	MW602102	MW602007	MW602039	MW602070
*Paraphlomishsiwenii* Y.P. Chen & Xiong Li 1	W.H. Wu et al. DD426 (KUN)	Jingxi, Guangxi, China	OP605346	OP609841	OP609848	OP609855	OP609862
*Paraphlomishsiwenii* Y.P. Chen & Xiong Li 2	W.H. Wu et al. DD426 (KUN)	Jingxi, Guangxi, China	OP605347	OP609842	OP609849	OP609856	OP609863
*Paraphlomisintermedia* C.Y. Wu & H.W. Li	X. Zhong et al. ZX16823 (CSH)	Suichang, Zhejiang, China	MW602135	MW602105	MW602010	MW602042	MW602073
Paraphlomisjavanica(Blume)Prainvar.javanica 1	Y.P. Chen s.n. (KUN)	Kunming, Yunnan, China	MW602121	MW602088	MW601993	MW602025	MW602057
Paraphlomisjavanica(Blume)Prainvar.javanica 2	L.B. Jia et al. JLB0029 (KUN)	Maguan, Yunnan, China	MW602143	MW602113	MW602017	MW602049	MW602080
Paraphlomisjavanicavar.pteropoda D. Fang & K.J. Yan	X. Li 2020090501 (GXF)	Jingxi, Guangxi, China	MW602140	MW602110	MW602015	MW602047	MW602078
*Paraphlomisjiangyongensis* X.L. Yu & A. Liu 1	A. Liu et al. LK1104 (CSFI)	Jiangyong, Hunan, China	MW602128	MW602095	MW602000	MW602032	MW602064
*Paraphlomisjiangyongensis* X.L. Yu & A. Liu 2	A. Liu et al. LK1104 (CSFI)	Jiangyong, Hunan, China	MW602129	MW602096	MW602001	MW602033	MW602065
*Paraphlomiskwangtungensis* C.Y. Wu & H.W. Li	Y.P. Chen & Y. Zhao EM1391 (KUN)	Huaiji, Guangdong, China	MW602126	MW602093	MW601998	MW602030	MW602062
*Paraphlomislanceolata* Hand.-Mazz. 1	C.Z. Huang s.n. (KUN)	Guidong, Hunan, China	MW602145	MW602115	MW602019	MW602051	MW602082
*Paraphlomislanceolata* Hand.-Mazz. 2	A. Liu et al. LK0825 (CSFI)	Ningyuan, Hunan, China	MW602146	MW602116	MW602020	MW602052	MW602083
*Paraphlomislancidentata* Y.Z. Sun	X. Zhong et al. ZX16824 (CSH)	Suichang, Zhejiang, China	MW602136	MW602106	MW602011	MW602043	MW602074
*Paraphlomislongicalyx* Y.P. Chen & C.L. Xiang	Y.P. Chen et al. EM583 (KUN)	Huanjiang, Guangxi, China	OK104771	OK104774	OK104778	OK104780	OK104783
*Paraphlomismembranacea* C.Y. Wu & H.W. Li	M.S. Nuraliev 1057 (MW)	Thanh Son, Phu Tho, Vietnam	/	MW602100	MW602005	MW602037	/
*Paraphlomisnana* Y.P. Chen, C. Xiong & C.L. Xiang 1	C. Xiong XC21097 (KUN)	Chengkou, Chongqing, China	OM836062	OM884451	OM884454	OM884457	OM884460
*Paraphlomisnana* Y.P. Chen, C. Xiong & C.L. Xiang 2	C. Xiong & H.L. Zhou XC21126 (KUN)	Wushan, Chongqing, China	OM836063	OM884452	OM884455	OM884458	OM884461
*Paraphlomispagantha* Dunn	L.X. Yuan et al. s.n. (KUN)	Qionghai, Hainan, China	OP605345	OP609840	OP609847	OP609854	OP609861
*Paraphlomispaucisetosa* C.Y. Wu 1	X.X. Zhu s.n. (KUN)	Malipo, Yunnan, China	MW602125	MW602092	MW601997	MW602029	MW602061
*Paraphlomispaucisetosa* C.Y. Wu 2	X. Li LX200704 (GXF)	Napo, Guangxi, China	MW602133	MW602103	MW602008	MW602040	MW602071
*Paraphlomisreflexa* C.Y. Wu & H.W. Li	Z.Z. Yang et al. s.n. (HIB)	Tongshan, Hubei, China	MW602122	MW602089	MW601994	MW602026	MW602058
***Paraphlomisyingdeensis* W.Y.Zhao, Y.Q.Li & Q.Fan 1**	**Q. Fan et al. 19013 (SYS)**	**Yingde, Guangdong, China**	**OP605348**	**OP609843**	**OP609850**	**OP609857**	**OP609864**
***Paraphlomisyingdeensis* W.Y.Zhao, Y.Q.Li & Q.Fan 2**	**Q. Fan et al. 19013 (SYS)**	**Yingde, Guangdong, China**	**OP605349**	**OP609844**	**OP609851**	**OP609858**	**OP609865**
***Paraphlomisyingdeensis* W.Y.Zhao, Y.Q.Li & Q.Fan 3**	**Q. Fan et al. 19013 (SYS)**	**Yingde, Guangdong, China**	/	**OP609845**	**OP609852**	**OP609859**	**OP609866**
*Phlomisfruticosa* L.	Y. Tong s.n. (KUN)	Shanghai, China (cultivated)	MW602119	MW602086	MW601991	MW602023	MW602055
Phlomoidesdentosavar.glabrescens (Danguy) C.L. Xiang & H. Peng	Y.P. Chen EM360 (KUN)	Beijing, China (cultivated)	MW602120	MW602087	MW601992	MW602024	MW602056
